# Adapting to Changes in Communication: The Orbitofrontal Cortex in Language and Speech Processing

**DOI:** 10.3390/brainsci14030264

**Published:** 2024-03-08

**Authors:** Xiaoming Jiang, Xiquan Ma, Ryan Sanford, Xun Li

**Affiliations:** 1 Institute of Linguistics and Key Laboratory of Language Sciences and Multilingual Intelligence Applications, Shanghai International Studies University, Shanghai 201620, China; xunli_heureux@163.com; 2 Department of Developmental and Behavioral Pediatrics, Shanghai Children’s Medical Center, Shanghai Jiao Tong University School of Medicine, Shanghai 200127, China; mxq919@163.com; 3 Department of Biomedical Engineering, McGill University, Montreal, QC H3A 2B4, Canada; ryan.sanford@mail.mcgill.ca

**Keywords:** uncinate fasciculus (UF), OFC, perisylvian network, social cognition, neurobiology of language

## Abstract

Despite most studies on the neurobiology of language demonstrating the central part of the perisylvian network involved in language and speech function, this review attempts to complement this view by focusing on the role of the orbitofrontal cortex (OFC). This region is primarily involved in goal-directed adaptive behavior. Recently, there has been increasing evidence that the OFC is involved in language and speech tasks. This review demonstrates that not only the linguistic tasks that involve the processing of socially, pragmatically and emotionally relevant information engage OFC and its neurobiological mechanisms, but also specific receptive and expressive language performances rely on specific neurophysiological properties of this region (e.g., the gray matter volume and the functional activation of OFC and the uncinate fasciculus that connects OFC), which in many cases, demand executive functions. These findings highlight: (1) The OFC plays a relevant role in the adaptive neurobiological function of language; (2) the neurobiological mechanisms beyond linguistic and speech processes complement and interplay with the language-unique processes to achieve successful comprehension and production in the changing communicative contexts.

## 1. Introduction

Language is created to serve human social function and is crucial for human adaptation to communicative settings or communicative goals [1]. One traditional view, which sees language processing as driven by domain-specific operations, argues that the language function is supported by the left perisylvian network, and early research on the language connectome mainly focused on the arcuate fasciculus that connects Broca’s and Wernicke’s Areas. However, recent work has shown that neural networks beyond language processing may support language and speech tasks in a broader and more communicative sense, proposing an insufficient role of traditional language networks underlying the language tasks and that domain-general networks, or neural networks serving other cognitive functions, such as executive and social functions, play crucial roles in certain language functions [2,3,4,5,6,7,8,9,10].

One possible candidate is the orbitofrontal cortex (OFC), which has been typically confirmed as functioning in adaptive processes in learning, memory and decision-making [11,12,13,14] for goal-directed behavior [15,16,17]. The OFC has been assigned a role in a neural circuit that supports the inference or mental simulation of consequences of novel experiences or encodes the change status in learned behaviors [18,19,20]. The medial OFC (mOFC) has been associated with the down-regulation of reward on pain perception [21], emphasizing a role of value comparison, which further guides socioeconomic choice behavior [22]. The lateral OFC causally influences fear regulation under uncertainty, resulting in a propensity for drinking behavior [23]. The OFC is arguably a region which allows associative learning, permitting value-related information, to be manipulated in representational memory and generating expectancies to influence downstream limbic areas for emotion and other prefrontal regions for guiding goal-directed behaviors [24].

Given these observations, the early proposal from these cognitive neuroscientists has shed light on the important role of OFC in adaptive behaviors, and efforts have seldom focused on language and speech, which have undoubtedly evolved for humans to survive in changing environments [1,25]. However, earlier studies, solely interested in the social-adaptive functions of the OFC, seem to show no functional association between OFC and language/speech functions. Such assumption could be primarily derived from neuropsychological studies, which intended to uncover the cognitive and behavioral consequences of those who suffer a particular OFC “damage” and reported above-average language and speech function regardless of whether the function of OFC is intact or not [26,27,28,29,30,31,32,33]. Recently, however, there has been increasing functional and structural neuroimaging evidence. This demonstrates that the OFC most likely plays a substantial role in human language functions, particularly for language tasks involving socio-emotional components [34,35,36,37,38,39,40,41,42].

In this review, we would like to respond to the appeal for a neural architecture that supports functions beyond what traditional perisylvian networks do in association with language and speech processing and, most importantly, to make a novel effort to extend what we know about OFC in social cognition to language functions. On the one hand, despite some anatomical link of OFC with the perisylvian frontotemporal linguistic network, the functional dichotomy of OFC and these regions is established (in humans: [43]; in macaque monkeys: [44]), emphasizing the role of OFC in mediating the relation of language processing through socio-emotional processes. On the other hand, the neurobiological mechanisms underlying OFC can participate in linguistic and speech-related functions on its own, as well as in coordinating and executing multiple processes in both the perception and the production of language.

## 2. Functional and Anatomical Characteristics

### 2.1. Functional and Anatomical Connectivity of OFC and Language-Related Regions

The OFC can be anatomically divided into medial and lateral subregions and anterior and posterior subregions. The cellular organization of OFC follows a gradient pattern along the anterior–posterior axis, with the posterior regions being agranular, the middle regions dysgranular, and the anterior regions fully granular. The OFC receives highly processed sensory information, including that which encodes bodily states and that from areas that process high-level emotional and social information. The medial and lateral OFC (lOFC) are considered as encoding stimuli of different valence or different sources of values (e.g., from external stimuli or the structure of the task, [45]). The OFC generates outputs to the medial prefrontal cortex, medial striatum and mediodorsal thalamus, which allows this region to encode associations between sensory stimuli in the external world and internal states and to send signals to be further integrated into ongoing higher-order cognitive operations in other parts of prefrontal regions [46].

The functional partitions have been defined within the OFC. A resting-state fMRI study based on 654 participants showed functional connectivities between the lOFC and the pars triangularis and pars opercularis (BA 44 and 45; regions involved in language processing as part of Broca’s area), the premotor cortical areas and the angular and supramarginal gyri, providing anatomical routes that link OFC with language functions [47]. A meta-analytic connectivity modeling study revealed coactivation of lOFC with the left IFG and left frontal operculum, regions primarily driven by studies involving semantic monitoring and discrimination, and the left STG and MTG, regions engaged in judgments of semantic plausibility and naming [48]. This finding suggests semantic processing areas in the prefrontal cortex extend further ventrally. The lOFC here has functional connections with the language-processing regions.

The OFC is anatomically linked via the ventromedial connection to the Broca’s area (lateral IFG; the pars orbitalis of BA 47; [49]). Such anatomical connectivities of OFC to language networks have functional implications in tasks. The cognitive inputs at the language level have been demonstrated to bias reward representations of odor, taste and flavor in the mOFC [50], with the stimuli of different linguistic labels activating OFC at different levels. The subjective pleasantness of rewarding stimuli (including taste, flavor and somatosensory ones) is also linearly related to the activations of the OFC when the pleasantness is influenced by linguistic labels [51,52,53], possibly due to the underlying anatomical link between OFC and language networks.

### 2.2. Functional Lateralization of OFC

The close examination of the cytoarchitectonical sub-regions within the lOFC revealed functional lateralization based on the meta-analysis [54]. The processing of linguistic information, which demands a high working memory, revealed activations in the left hemisphere. In contrast, the nonlinguistic processing, such as the perception of gustational input and physical pain, was in the right hemisphere. Two more lateral and more posterior subregions in the left hemisphere (Fo6 and Fo7 based on cytoarchitectonic characterization) showed activations in verbal fluency [55], semantic and orthographic processing in language tasks [56,57], in actions involving working memory, as well as emotion-driven cognitive processes. The mOFC may be more associated with general cognitive functions, such as cognitive slowing, which seems to be right-lateralized. The slower processing speed (measured with the NIH Toolbox Pattern Comparison Processing Speed Test) was associated with the decreased local gyrification in the mOFC in the right hemisphere [58].

What is the link between the lateralization of OFC and language deficits? The OFC is considered a part of the anatomic network underlying the hemispheric language dominance (HLD), the cerebral hemisphere, most dominant for expressive and receptive language function. Rightward asymmetry was found in OFC in epileptic patients with left or right HLD, although such asymmetry was stronger in patients with right HLD [59].

The altered lateralization of the OFC has been observed in autism spectrum disorder. ASD patients typically demonstrated reduced leftward language lateralization (for example, the lower leftward volume asymmetry of language-related regions such as the planum temporale, Heschl’s gyrus, posterior supramarginal gyrus and parietal operculum) relative to the controls [60]. In addition, the altered asymmetries of cortical thickness in ASD were demonstrated in medial and lOFC relative to controls, and the asymmetry of the surface area of the OFC was also reduced [61]. These reduced asymmetries may be related to the disrupted executive functions in ASD and maybe involved in difficulties in resolving linguistic conflicts and repetitive and stereotyped behavior in this special population. The language deficits in people with ASD may be due to altered lateralization of the OFC, but it is also likely that ASD causes the altered lateralization of the OFC and the language deficits. The loss of gray matter volume (GMV) in the hippocampal gyrus was correlated with the structural alterations in OFC [62]. The OFC subserves the reorganization of language lateralization (from left to right) for left TLE patients.

## 3. Methods

Below, we review studies on neuroimaging and neuropsychological approaches to language and speech. The literature included in the review was searched with the key words combinations (i.e., “OFC” and “language”, “OFC” and “speech”, “orbitofrontal cortex” and “language”, and “orbitofrontal cortex” and “speech”) on Web of Science, PsycINFO and Google Scholar, which were published between 1990 and 2020. The starting year was determined based on an initial search with an unspecified time range, which showed that the research that reported the involvement of OFC in language/speech processing tasks had started to evolve. Mendeley was used to manage and deduplicate search results. We limited the reviewed literature to that using neuroimaging recording and analysis techniques and reported peak coordinates associated with OFC (i.e., fMRI, MEG, fNIRS, sMRI, EEG source localization, ECoG). As for the research question, the studies included in the review must directly test the neural activation of language or speech processing and/or tested language or speech-relevant performances and correlate them with the structural/functional characteristics of the brain. Most eligible studies were conducted on healthy adults, but a small portion was conducted on patients, ageing populations or infants, or bilingual individuals. To keep the data homogeneous and the review’s findings more focused, we only kept studies on healthy adults. Studies which expressed explicit interest in bilingual language/speech processing were also not included in the review (Table 1, Table 2 and Table 3). All studies have shown the involvement of some portions of the OFC in certain language or speech tasks.

The initial search rendered 307 studies. The exclusion of those without focusing on language/speech processing or testing language or speech performances and correlating them with structural/functional brain characteristics resulted in 186 studies. Reviews were further excluded, rendering 151 studies. The last round of exclusion of studies on special populations and neuroimaging studies that did not report a coordinate for OFC resulted in 43 studies.

**Figure 1:**
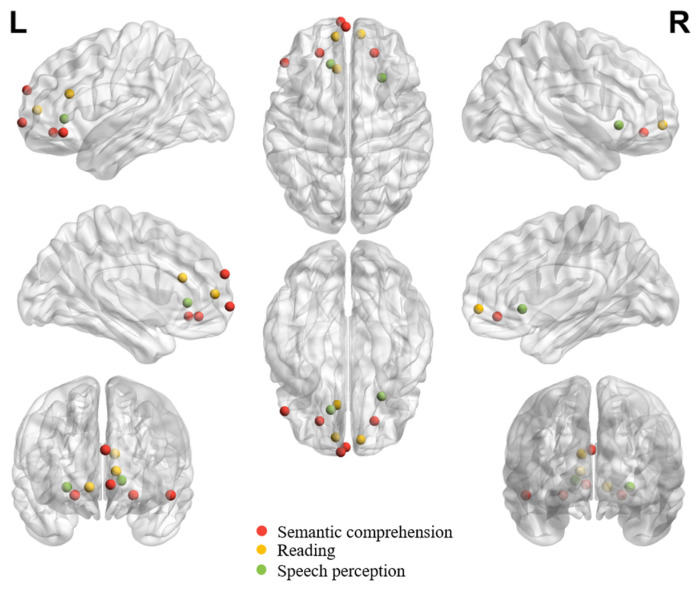
Individual peaks from neuroimaging studies on receptive language performance in healthy adults, including studies on **semantic comprehension** (number of coordinates = 5; [63]: (−6, 66, −2); [63]: (−2, 62, 24); [64], 2011: (−23, 41, −10); [64]: (21, 41, −10); [65]: (−51, 33, −10)), studies on **reading** (number of coordinates = 3; [66]: (−9, 28, 21); [67]: (10, 56, −4); [67]: (−10, 54, 8)), and studies on **speech perception** (number of coordinates = 2; [68]: (−14, 32, 1); [69]: (27, 21, −4)), are shown together according to their peak coordinates in MNI coordinate system.

**Figure 2:**
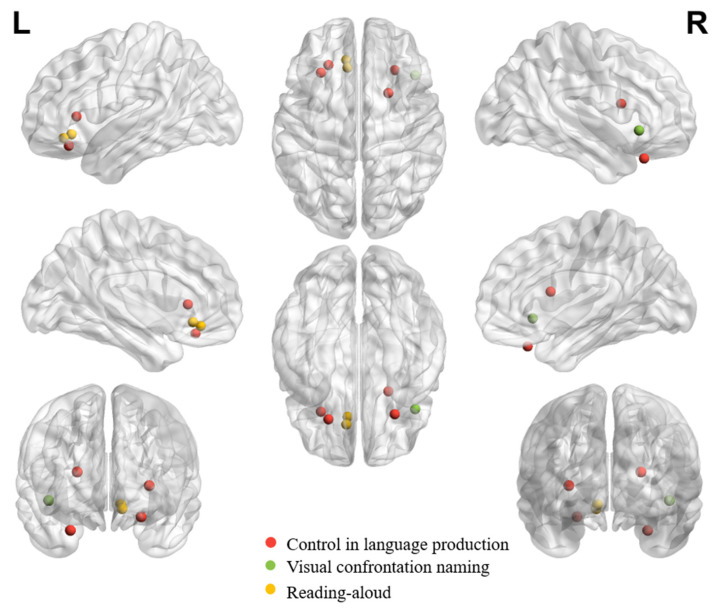
Individual peaks from neuroimaging studies on expressive language performance in healthy adults, including the studies on **control in language production** (number of coordinates = 4; [70]: (28, 28, −28); [71]: (−24, 32, −18); [72]: (23, 10, 15); [72]: (−30, 26, 5)), studies on **visual confrontation naming** (number of coordinates = 1; [73]: (44, 24, −6)), and studies on **reading aloud** (number of coordinates = 2; [74]: (−9, 30, −9); [75]: (−10, 36, −12)) are shown together according to their peak coordinates in MNI coordinate system.

Table 1, Table 2 and Table 3 summarize all eligible neuroimaging studies that reported peak coordinates in orbitofrontal regions in healthy adults in different tasks or processes. We focused on these eligible literature during the review. We still referred to other neuroimaging studies, neuropsychological studies or review papers whenever necessary to facilitate understanding the role of OFC in different tasks/functions. Tasks, languages, findings and functions of interest of all eligible studies are listed in the tables. The tables list studies in chronological order to facilitate tracking evolution trends. While the tasks of interest can be systematically synthesized according to (1) the receptive (Table 1), (2) the expressive (Table 2), and (3) the socially, pragmatically, and emotionally relevant language processing functions (Table 3), the locations of peak activations in the OFC are distributed broadly across tasks and functions. To gauge the potential distribution pattern within OFC regarding adaptive functions in speech and language communication, we visualize individual peaks from neuroimaging studies on receptive language (Figure 1) and expressive language performance (Figure 2) in healthy adults. The peaks in OFC are more anteriorly and dorsally distributed when observed in receptive language than expressive language tasks, despite the peaks being bilaterally distributed regardless of the nature of the task.

## 4. OFC and Receptive Language Performance

### 4.1. Semantic Processing in Language Comprehension

The OFC is primarily involved in semantic processing, consisting of other complex cognitive processes, including semantic coercion, prediction, inference, thematic processing, multimodal semantic processing, etc. As is shown in Table 1, an enhanced activity in OFC can be found in semantic relationships which are ambiguous, incongruent, or difficult to integrate or in tasks that demand additional resources or executive efforts to check for semantic congruency.

The left mOFC appears to be involved in semantic composition, the process of coercing meanings of sentential constituents to match those with a specific constraint, for example, the aspectual coercion in which the prepositional phrase “for an hour” coerces the lexical meaning of “deliver the meals” to be iterative across the entire duration [105,106,107]. In one study, when contrasting coercion expressions (e.g., the journalist began the article, in which the object complement was coerced into an underspecified event meaning) with noncoercive control sentences (e.g., the journalist wrote the article, in which no such coercion was needed given the verb explicitly stated the event meaning) during an MEG recording, increased activity was observed for coercion in a prefrontal midline field, which can be localized in the OFC [105]. No such effect was found for implausible control sentences, suggesting the unique role of OFC in the computation of coercion and not in the detection of poor real-world fit.

The OFC was associated with the cross-modal integration of semantic information. The MEG recorded the audio–visual stimuli, which comprised a simultaneously presented image depicting an animal or object accompanied by the corresponding sound (e.g., a picture of a bird paired with the sound of chirping). The matched pairs of picture and sound elicited a more significant response after the onset of the audio–visual stimuli between 400 ms and 1000 ms in bilateral OFC compared with the mismatched pairs, confirming the role of integrating multisensory linguistic information in the OFC [64].

Moreover, the OFC maybe involved in the decision making process during lexical ambiguity resolution. Reading sentences that included a pronoun, the antecedent of which was clearly indicated in the context, activated the left OFC compared to those in which the antecedent of the pronoun can only be inferred and those in which the antecedent of the pronoun was ambiguous. Moreover, the uncertainty of ambiguity resolution was correlated with the activity in the bilateral OFC. Sentences with ambiguous pronouns in their referring antecedents revealed increased activation in OFC compared to those of unambiguous reference or of no appropriate antecedent [63]. The OFC is engaged in the top-down evaluation of the risk associated with making an uncertain linguistic choice [65]. Notably, the OFC may serve as a neural mechanism related to linguistic decision making independent of the perisylvian network that supports syntactic and semantic resources for interpreting referential meaning in sentence comprehension.

The lexico-semantic prediction has been associated with the functional connectivity between the lOFC and other frontotemporal regions. With magnetoencephalography (MEG) and a sentence reading paradigm, the target noun’s predictability varied given its context in simple German sentences. Increased functional connectivity was found at 400 ms between left lOFC and right IFG within the beta band and from 300 to 700 ms between left OFC and left STG within the beta and low gamma bands, on less predicted nouns relative to highly predicted nouns. The increased intertrial phase-locking value (measured through the consistent phase difference between regions across trials) to a weakly predicted item in the beta band between the left lOFC and STG may represent a top-down impact on lexical retrieval [108]. These findings suggest the role of OFC in building contextual prediction towards a lexical representation [109,110].

The OFC has been shown to be involved in processing thematic constraints (revealing who did what to whom). An MEG study on Greek morphological processing revealed differential activity in OFC peaking around 300 ms and spanning until 500 ms between the combination of stem and suffix, which violated the compositional semantic rule (e.g., in argi-menos, -menos requires a transitive verb, while argi is intransitive) vs. the combination violating categorical constraint (e.g., in ahino-menos, -menos requires a verb, while ahino is a noun; [111]). This finding confirmed the critical role of OFC in semantic word- and sentence-level processing [106,112,113].

The OFC is reported to be associated with the semantic fit between stems and affixes in complex morphological words. An MEG study showed that the corpus-based semantic coherence measure of the gradient semantic fit of stems and affixes was correlated with the left OFC activity in the 350 and 500 ms [112]. Moreover, in a lexical decision priming study, the activity in the left OFC was reduced when the prime and the target words shared morphological and semantic features (e.g., ourson_prime_−ours_target_/cup bear_prime_−bear_target_), compared to when no association existed (e.g., oursin_prime_−ours_target_/urchin_prime_−bear_target_) or only orthographic features were shared in French (e.g., gésier_prime_−ours_target_/gizzard_prime_−bear_target_; [114]). These findings highlight the role of OFC in the semantically driven morphological recombination stage, where morphemic units are recombined to recognize the whole word.

The OFC is shown to serve a top-down impact on single-word comprehension. With MEG, neural responses towards nouns and verbs preceded by a predictive (possessive pronouns for nouns and personal pronouns for verbs, e.g., your bag vs. you take) or a nonpredictive syntactic context were tracked. Sentences with possessive and personal pronouns differed in source areas, including bilateral mOFC, with the possessive pronoun eliciting a stronger source activity than the personal pronoun post-onset of pronouns [115]. Such activation is related to the domain-general network in the proactive processing language and may exert a top-down modulation towards other linguistic perisylvian regions, such as the left IFG.

The relation of the OFC with inferential processing has been shown in an fMRI study. The activity in the OFC was enhanced when the listener attended to dialogue when presented audio–visually, as compared with the listener who attended to the fixation cross but ignored the dialogue [116]. The most likely roles the OFC undertakes in the former task are processing semantic contents and making inferences about the interlocutor’s characteristics and social attributes in the dialogue. Many inferential tasks involve semantic control. The OFC may engage in semantic control, which is crucial to forming organized thoughts.

### 4.2. Reading

The OFC is relevant to efforts in reading. As shown in Table 1, enhanced activity in OFC can be seen when reading tasks become increasingly complex, demand more attentional resources, or when individuals show reduced executive resources for the reading task.

When sentences were visually presented at a faster rate, an increased phase synchronization was shown between OFC and occipitotemporal reading-related regions and between OFC and superior temporal gyrus responsible for semantic and phonological analysis, compared with a slow presentation rate [66], suggesting the role of OFC in visual processing and linguistic readings.

Reading comprehension performance is associated with the OFC. Young adults with a higher reading ability (as measured with the Woodcock Reading Mastery Test–Revised, WRMT-R, [117]) revealed decreased activation in the orbital frontal gyrus. The mOFC activity was less pronounced for those who exhibited higher executive function during reading and those who completed an n-back task more efficiently [67]. These findings implicate that the OFC may function as the general cognitive processes underlying reading, which allows information processed during reading tasks to be linked more efficiently with previously acquired information.

### 4.3. Speech Perception

The OFC is involved in speech processing. The level of OFC involvement can be modulated by attentional engagement, such as the listener’s strategies and capabilities to focus on speech. The tasks that demand more attentional resources engage a higher level of OFC (Table 1). The left OFC was more activated when participants heard speech relative to reversed-speech conditions, and such activation was more pronounced when they focused on listening to the sound rather than ignoring such stimuli. Moreover, the enhanced left OFC activity was found only in words rather than pseudowords when the listeners attended to the stimuli [68].

The mOFC is associated with one’s capacity to fill out irrelevant information in speech recognition. The OFC is involved in executive processes in dealing with phonetic change. In an auditory oddball paradigm, the deviant stimuli (tone one or tone two) elicited a magnetic counterpart of mismatch negativity (MMNm) compared to the standard one (tone 3) in an MEG study. When detecting deviant relative to the standard stimuli, the right OFC was activated at around 200 following the activation in the bilateral STG. These findings indicate that the right OFC reflects the detection of tonal change, which may trigger an update in the predictions about what sounds will likely be encountered in the near future [69].

The rhythmic neural activity in the OFC has been associated with predictive processing in speech comprehension. The OFC increased its power in the gamma band for the standard rather than deviant stimuli when listeners actively discriminate between auditory stimuli. At the same time, they ignored simultaneous videos during an oddball task [118], indicating the auditory input matched the speaker’s predictions. The delta entrainment (the alignment of the slow auditory delta band activity to the rhythmic fluctuations in speech) in the left anterior STG was correlated with the beta power increase in mOFC [119]. This finding is consistent with the idea that the OFC serves as top-down modulation on auditory encoding, and such a process can also be seen in the OFC band power.

The OFC was related to phonemic and lexico-semantic processes during speech comprehension. This cortical region for top-down processing of speech was engaged to support better listening task performance. In another study, the activation in the left OFC was predicted by the interaural accuracy difference in the dichotic listening test across listeners when they repeated the syllables heard in each ear in individuals with unimpaired hearing but not those with listening difficulties [120].

In sum, these studies highlight the common mechanisms of how OFC is involved in receptive language performances. To achieve a specific goal in language and speech comprehension, the OFC is implemented by readers or listeners to aid in generating predictions about certain lexical stimuli for semantic comprehension and coordinating multiple resources to ensure reading and speech perception where additional domain-general resources are demanded.

## 5. OFC and Expressive Language Performance

### 5.1. Control in Language Production

Although we did not limit the search to the executive function of OFC in language production, the findings demonstrate that in tasks of speech or language production, the activation of OFC is related to the cognitive control demand in a variety of production tasks such as word conjugation, free recall verbal fluency, sentence production, conversation, etc. Different portions of the OFC have been implicated in previous studies of attentional set shifting and cognitive flexibility [121,122]. The OFC is involved in language control during sentence production. As is shown in Table 2, enhanced activity in OFC can be found in language production tasks that engage executive demands to coordinate simultaneous cognitive or linguistic operations.

During a Hayling Sentence Completion Task, participants were asked to complete the sentence with a semantically related or congruent word (in the initiation condition) or with a semantically unrelated or incongruent word (in the suppression condition) while undergoing MRI scanning. Enhanced activation of OFC was seen when the speaker continued a sentence with a weaker contextual constraint than a sentence with a stronger constraint [70]. Moreover, the left OFC activity was stronger in the incongruent suppression condition than in the congruent initiation condition [71]. The left OFC may be related to the generation of an incongruent alternative word or the inhibition of a congruent word in sentence completion. These findings suggest that the OFC showed a pattern of activation more in-line with the reactive control or conflict monitoring, which is demanded in the reactive task execution.

The OFC is also involved in the top-down modulation of executive demands in multi-task settings. The activation in the OFC was increased for a speaker during simulated driving when a conversation was engaged as compared with when no conversation was engaged. Increased activations were also found in language regions, which include Broca’s and Wernicke’s areas and bilateral IFG [72]. These findings showed that the OFC participated in the concurrent conversational task which engaged monitoring and executive functions.

The OFC may participate in regulating and monitoring speech to conform to the socio-communicative norm. Communicating with an actor with whom they usually spoke in a second language, instead of describing what an actor was doing in L2, the speakers showed OFC activity correlated with their anxiety levels and oral proficiency. The increased level of anxiety during speaking reduced activation in the OFC [123]. The OFC is involved in anticipating how speakers respond and monitoring whether their communicative utterances are appropriate with the interpersonal rules.

The activity in the left lOFC was enhanced during the verbal conjugation of a verb in a certain inflectional rule compared to during the preparation of choosing a language in which to generate this verb and choosing which inflectional morphology (plural vs. singular) to select to conjugate the verb, possibly related with the increased demand in working memory for the former compared to the latter task. Importantly, the increased OFC activity was found in trials when participants were not instructed with which morphological rule to conjugate the verb beforehand (reactive control) relative to those when they were instructed (proactive control)). Such an effect was positively correlated with the effect of reactive control vs. proactive control on the anterior cingulate cortex (ACC) and the IFG activity [124].

### 5.2. Naming

As is shown in Table 2, the OFC is more engaged during naming tasks when speakers are required to access the semantic memory of people or objects.

The OFC is involved in producing the proper name. DTI evidence showed that the integrity of UF was associated with a deficit in naming famous people, which is impaired the most in elderly patients [125]. This finding suggests the role of OFC in coordinating the encoding of faces and processing of famous names during name production, and this role can further explain the relation between the decrease in the integrity of UF and the tip of the tongue states (unable to retrieve a lexical term from memory, often accompanied with partial recall and the feeling of knowing) in older people.

The activity in the right OFC is correlated with the correctly named items in the visual confrontation naming task (VCN), in which the participants were asked to name the objects that were visually presented to them [73].

### 5.3. Reading Aloud

As is shown in Table 2, enhanced activity in the OFC can be observed when participants read aloud more familiar than unfamiliar words. At the same time, individual differences can also modulate the enhanced activation when accessing the mental dictionary of the lexical item is more demanding.

The left OFC can be associated with the semantic retrieval effort in the reading aloud task. The recorded Steady-State Visual-Evoked Potentials (SSVEP) revealed that the word frequency effect on the SSVEP activity in a reading aloud task could be localized in the left OFC, with higher activity in the high- than the low-frequency words [75]. The OFC activation was enhanced when participants were asked to read aloud words rather than nonwords, and such an effect was more pronounced as a function of age [74]. The findings point to a possible role of OFC in actively searching for a lexical item in the semantic network during language production [126].

To conclude, these studies showed the common mechanisms of OFC in executing cognitive monitoring or motor control in language and speech production during various expressive language tasks. Without the tact functioning of the OFC, impaired performances could exist in speech motor control, inhibition of disorganized thoughts, or inappropriate linguistic representation and naming.

## 6. Processing Pragmatically, Socially and Emotionally Relevant Information in Language Communication

**Figure 3:**
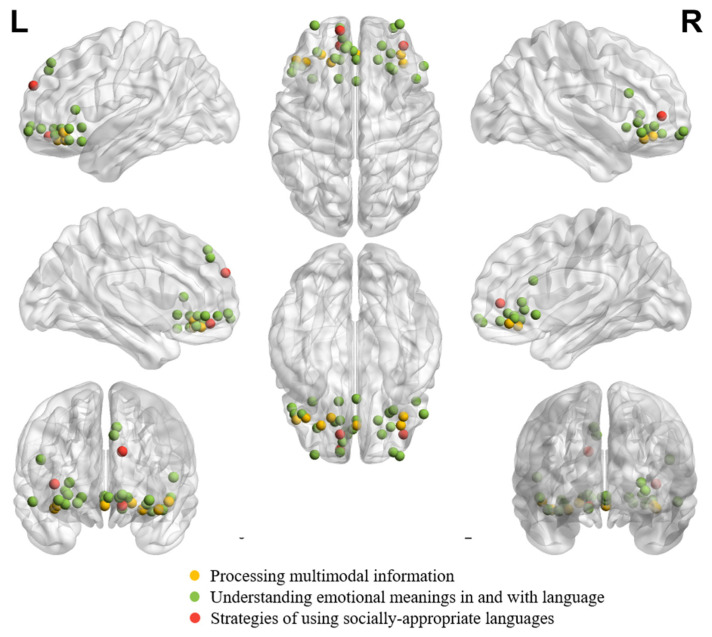
Individual peaks from neuroimaging studies in healthy adults on **processing multimodal information** (number of coordinates = 7; [76]: (1, 39, −12); [77]: (−19, 33, −10); [78]: (36, 39, −12); [79]: (−39, 33, −15); [80]: (38, 32, −14); [43]: (−27, 39, −15); [43]: (−46.5, 34.5, −9)), studies on **understanding emotional meanings in and with language** (number of coordinates = 25; [81]: (1, 18, −5); [82]: (−6, 46, 40); [82]: (−8, 44, 46); [83]: (−12, 60, −4); [84]: (−10, 52, −6)); [85]: (48, 20, 22); [86]: (−30, 41, −11); [87]: (−48, 30, −6); [88]: (27, 29, 5); [88]: (30,27,−1); [89]: (30, 60, −11); [91]: (−2, 42, −6); [92]: (54, 30, −9); [92]: (−42, 30, −15); [93]: (18, 35, −6); [93]: (−50, 25, 9); [94]: (21, 46, −10); [95]: (−16, 36, −6); [96]: (−32,62,−8); [96]: (35, 63, −8); [97]: (28, 40, −4); [98]: (−12, 35, −14); [99]: (1,35,−8); [100]: (−34, 20, −16); [101]: (−13, 20, −5), and studies on **strategies of using socially appropriate languages** (number of coordinates = 3; [102]: (−12, 46, −12); [103]: (−12, 58, 28); [104]: (38, 46, 4)) are shown together according to their peak coordinates in MNI coordinate. A result from a study was excluded from the figure (number of coordinates = 1; [90]: (45, 55, −5)), given that the peak coordinate was located outside the brain due to an EEG Source Localization Problem [127].

Figure 3 demonstrates individual peaks from neuroimaging studies on processing multimodal information, understanding emotional meanings in and with language, and strategies for using socially appropriate languages in healthy adults. It can be seen from Figure 3 that the peaks are bilaterally distributed across the study focuses. Moreover, the peaks from studies on communicators’ strategies of using socially appropriate and pragmatic felicitous languages are more anteriorly distributed than those from studies on multimodal information processing and emotional meaning understanding.

### 6.1. Processing Multimodal Information

A widely accepted view considers OFC a packaging centre that receives multimodal perceptual information from multiple sensory areas. As is shown in Table 3, enhanced activity in OFC can be seen in the face of multimodal linguistic inputs when access and integration of information in different modalities are demanded.

For example, story comprehension requires inputs from multimodal channels and engages OFC. The activity in mOFC increased as a function of the comprehensibility of the story provided by the listener, which could be contributed by the story’s coherence and the exposure of an additional picture preceding the story [76]. The OFC activation most likely reflects an emotional response to the reward of increasing comprehensibility during story understanding.

The OFC was associated with the recognition of the communicative act across modalities. In one fMRI study, the participants judged the match of a novel pairing of a sound and a communicative hand gesture. The right OFC activity was negatively associated with an improvement in learning performance, such that the reduced activity showed greater improvement in the judgment task [78].

The primary olfactory region in the OFC is suggested to have connections to the language network, which can explain how olfactory perception and language function may share neural mechanisms. Therefore, it is sometimes tricky when individuals name odors, and one’s flavor perceptions and flavor preferences are biased by linguistic labels [128]. The OFC was associated with the processing of multisensory information that was activated by words. For example, the increased OFC activity was also observed when participants verified words on the knowledge of taste [77], reflecting the retrieval of sensory-related knowledge during word recognition. Words related to edible items or with taste and flavor properties led to increased activation of the OFC that is associated with the processing of olfactory and gustatory sensations [77] even when no explicit gustatory judgment is involved, and participants read these words passively (within the left lOFC, [79]). In the fMRI adaptation study, words preceded by semantically matched odor revealed habituation in the right OFC and no such effect was found for those preceded by visual objects [80]. This finding suggests the modality-specific role of OFC in integrating cross-modal information and its role in working memory maintenance and episodic memory encoding due to the verbal translation of olfactory cues. The OFC is most likely involved in integrating lexical and sensory information in these studies.

The role of lOFC in speech–face integration was implicated in functional near-infrared spectroscopy (fNIRs), fMRI, and diffusion tensor imaging (DTI) studies. A study used the fMRI adaptation paradigm in which participants judged emotions from face and voice and found that the repeated exposure resulted in habituation in the OFC regardless of the linguistic modality. A DTI study revealed separable fiber projections from STS to OFC, including an external capsule for processing voice and dorsal superior longitudinal fasciculus (SLF) for processing face and ventral dorsal SLF for integrating voice and face [129]. This finding is consistent with the view that the OFC constitutes an interface linking differential communicative signals with regions that guide behavioral responses toward an integral linguistic representation during communication. In the fNIRs study, the activity in OFC-relevant channels was reduced when participants viewed a computer-generated face compared to a real face. Moreover, the lOFC activity was enhanced when the computer-generated face was accompanied by speech prosody relative to the muted face. It is suggested that the lOFC is sensitive to the motivational (rewarding/punishing) implications associated with speech prosody, leading to increased appraisal response [130].

The OFC has also been shown to be related to perceptual decision making based on sensory knowledge. Moreover, the OFC facilitates semantic processing during reading lexical olfaction. Compared with reading literal paraphrases (He cannot stand him at all), reading olfactory metaphors (He cannot smell him at all) and literal olfactory sentences (He smells very unpleasant) activated the left OFC (the secondary olfactory cortex) as well as perisylvian networks [43]. This observation supports the action–perception theory of semantic circuits [131], which predicts that reading verbs associated with smelling can evoke odor-related emotional responses to the words. These findings indicate the executive role of left OFC in selecting task-relevant information (emotional connotations) from competing semantic alternatives. These findings suggest a possible role of OFC in acquiring communicative meaning via multimodal channels.

### 6.2. Understanding Emotional Meanings in and with Language

**Figure 4:**
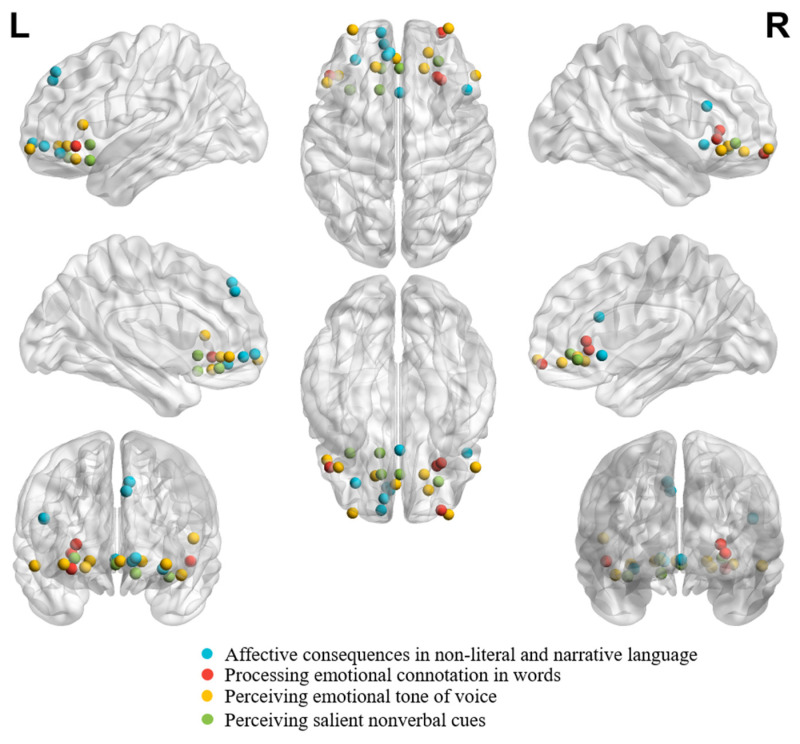
Individual peaks from neuroimaging studies in healthy adults on **affective consequences in non-literal and narrative language** (number of coordinates = 7; [81]: (1, 18, −5); [82]: (−6, 46, 40); [82]: (−8, 44, 46); [83]: (−12, 60, −4); [84]: (−10, 52, −6)); [85]: (48, 20, 22); [86]: (−30, 41, −11), studies on **processing emotional connotation in words** (number of coordinates = 4; [87]: (−48, 30, −6); [88]: (27, 29, 5); [88]: (30, 27, −1); [89]: (30, 60, −11)), studies on **perceiving emotional tone of voice** (number of coordinates = 9; [91]: (−2,42,−6); [92]: (54, 30, −9); [92]: (−42, 30, −15); [93]: (18, 35, −6); [93]: (−50, 25, 9); [94]: (21, 46, −10); [95]: (−16, 36, −6); [96]: (−32, 62, −8); [96]: (35, 63, −8)), and studies on **perceiving salient nonverbal cues** (number of coordinates = 5; [97]: (28, 40, −4); [98]: (−12, 35, −14); [99]: (1, 35, −8); [100]: (−34, 20, −16); [101]: (−13, 20, −5)). Studies on are shown together according to their peak coordinates in MNI coordinate. A result from a study was excluded from the figure (number of coordinates = 1; [90]: (45, 55, −5)), given that the peak coordinate was located outside the brain due to the EEG Source Localization Problem [127].

Figure 4 demonstrates individual peaks from studies on affective consequences in non-literal and narrative language, processing emotional connotation in words, perceiving emotional tone of voice, and perceiving salient nonverbal cues.

It can be seen from Figure 4 that peaks are bilaterally distributed across study focuses. Moreover, the peaks from studies on affective consequences in non-literal and narrative language appear more superiorly distributed.

#### 6.2.1. Affective Consequences in Non-Literal and Narrative Languages

As shown in Figure 3, enhanced activation of OFC is expected to occur when comprehenders process narratives, jokes, and stories that induce highly affective experiences or in tasks that require one to explicitly evaluate the socio-affective value of the language (Table 3).

The OFC may interact with regions of semantic memory and use the positive and negative outcome memory associated with the language under different contexts to guide the interpretation of non-literal meaning in a social context. Impairments in the identification of sarcasm and emotion in video vignettes are associated with atrophy in the lOFC [132], suggesting that some deficits in OFC are associated with the failed use of social knowledge that involves the understanding of language as a context-appropriate behavior [133].

During narrative comprehension, the OFC is also associated with building emotional aspects in the situation model. The OFC was activated when listeners heard a story in which the protagonist’s emotional state (e.g., happy or sad) was described, as compared with those without these descriptions [81]. This activation is associated with the listener’s empathy towards the story’s character when evaluating emotional information in story comprehension. The OFC is a part of the network that supports the pleasure experienced during narrative hearing [134]. The OFC is also involved in the moral and social judgments towards the protagonist’s behavior described in a story. The lOFC was more activated when participants read stories and judged whether the character’s behavior served a moral purpose or was telling a lie, as compared with when they judged the gender of the character [82]. The OFC tracks valence information in the auditorily presented narratives. The OFC was activated when listeners were engaged to hear stories with positive valence [83]. Moreover semantic events with negative valence were associated with increased inter-subject phrase synchronization connectivity in bilateral OFC. In contrast, positive valence was associated with increased activity in OFC and primary somatosensory cortex [84]. Increased functional connectivity of OFC and parietal/occipital mentalizing areas was found when readers rated narrative texts to be more suspenseful [85]. These findings indicate the role of OFC in tracking speech-based emotional information.

The OFC aims to support the evaluation of affective consequences from the interpretation of nonliteral meaning. Reading jokes that demand inferences towards their contents or the targets (the bridging inference type) activated the left OFC compared to their literal counterparts [86]. The OFC plays a mediating role in coordinating the relationship between the social, affective information and socially oriented intentions for the inferred contents, therefore rendering felt experience that arises from the interpretation of the jokes.

#### 6.2.2. Processing Emotional Connotation in Words

It is not possible to use words without communicating emotional connotations or to understand language based on denotation alone [135]. The Quartet Theory has predicted a direct link between the OFC and word processing. Koelsch argues that both written and spoken words can be recognized by an affective system even before the processing in the language network, meaning that the words can obtain symbolic quality (which can be learned through contexts) by which they can elicit affective activity in the OFC [136]. The OFC-based judgments reflect the emergence of a felt sense towards a word that precedes the articulation of the nameable category to which it belongs. Though this theory does not attribute language processing per se to the function of OFC, it emphasizes the crucial role of the OFC in building associations between linguistic representation and emotional meanings [137].

Building on this theory, evidence has associated the OFC with the semantic judgment of the emotional connotations in words. As is shown in Table 3, an enhanced activity in the OFC can usually be for linguistic stimuli with positive vs. negative valence or those with weaker negative meanings, which indicate that the OFC appears to be related to one’s emotional regulation of negative emotion.

The OFC responds to the positive and negative valence of words such as yes and no. An fMRI study in which participants judged words and sub-vocally expressed the words revealed faster reaction times and increased activity in OFC for yes and slower response times and decreased activity in the right lOFC for no, compared with their relevant baselines. Moreover, the more significant activity induced in the OFC, the more negative valence was attributed to no, and greater anger control was applied to this word [88].

The lOFC is sensitive to the specific meanings of words and forms a symbolic representation of social values (such as reward and punishment), consistent with the anatomical link between lOFC and visual and auditory areas. The functional and anatomic evidence supports the involvement of lOFC in response to auditory and visual language stimulation. The left lOFC was more activated when listeners were asked to judge the semantic attributes of adjectives that convey emotional meanings than when they judged the affective prosody intoned in the adjectives [87]. Using combined EEG/MEG, the activated structures underlying the positive modulation of positive vs. negative words (between 150 and 190 ms; P2) showed the peak in the right middle OFC [89], suggesting the role of OFC in mediating the possible rewarding value underlying the positive-valenced words. Moreover, using EEG, the early modulation of positive vs. negative words (at around 120 ms; N1) can be localized to the OFC [90], highlighting the role of the OFC in quickly and automatically capturing attentional resources on positive words, diverting them from the task where the attention was voluntarily directed. Further evidence has suggested the role of OFC in distinguishing emotional meanings at the word level.

Sex differences in the frequency of using strong swear words are correlated with differential volumes of OFC in males and females. Females generally have larger volumes of OFC and a larger OFC-to-amygdala ratio that modulate aggressiveness generated by the amygdala, and such differences influence the use of strong swear words [138]. During auditory–verbal memory, females were more likely than males to show increased activations in the OFC, which is associated with inhibitory functions.

#### 6.2.3. Perceiving Emotional Tone of Voice

Proponents link the OFC with the emotional perception in speech, particularly the tone of voice [139]. Enhanced activation of the OFC is typically associated with judging emotions from prosodically marked vs. unmarked speeches, emotional vs. neutral voices, or spoken words with conflicting emotional meanings from vocal and lexico-semantic dimensions (Table 3). Schirmer and Kotz’s model relates the OFC to the evaluative judgment of the emotionally significant information that is identified from the ventral stream, including STG and superior temporal sulcus (STS), suggesting the role of higher-level cognitive processing of lower-level vocal stimuli [140].

It is noted that the level of OFC involved in perceiving emotional speech prosody is associated with the perceived strength of consonance and/or the emotional responses towards such perception. Increased OFC activity was found for listening to singing, compared with listening to speech [91]. Neuroimaging studies have demonstrated increased bilateral OFC activity when listeners evaluated the emotional information rather than when they evaluated linguistic aspects of speech [141].

Moreover, the OFC has been associated with the detection of vocal information that is both novel and behaviorally relevant. In an fMRI study with a habituation design, the bilateral OFC showed more robust responses when participants were asked to classify vocal emotion compared to the word class of the vocal stimuli. It was more responsive to the anger expressed in the speech prosody. The bilateral OFC also showed selective habituation with an interaction between emotion and repetition, with particularly pronounced responses to angry prosody during the first presentation, prompting its role in evaluating and responding to linguistic stimuli with affective value when these have not been encountered before [92].

The involvement of the OFC in the detection of emotional prosody is subject to the task demand on executive control. Experimental blocks with more trials in which the lexico-semantic emotional cues conflicted with prosody showed higher activation in the OFC [93]. Enhanced OFC can also be observed when listeners were required to evaluate a “lack of credibility” from vocal expression rather than neutral voice [95]. The fNIRS study showed increased activity in the bilateral orbitofrontal region in speech intoned with angry prosody relative to neutral speech [96]. The OFC most likely plays a role in conflict resolution and suppression of inappropriate meaning associated with the tone of voice.

Despite the majority of evidence that links the OFC with executive demands in vocal emotion perception, some studies demonstrated that the connectivity of the right OFC (together with some subcortical regions, e.g., left subthalamic nucleus (STN)) plays a role in recognizing emotion from speech when the information is not task-relevant. The functional connectivity between the right OFC and left STN increased for emotional rather than neutral prosody when listeners judged gender from the speech. Moreover, the probabilistic fiber tracking corroborated such findings by showing the terminal localization of these fiber tracks in the right OFC and left STN [94].

#### 6.2.4. Perceiving Salient Nonverbal Cues

The OFC was associated with recognizing salient cues, such as vocal cues of particular relevance to the listener and dynamic body cues. Enhanced activation of OFC is typically expected to be associated with perceiving salient vs. non-salient cues from a nonverbal display (Table 3).

Firstly, the activity and functional connectivity of OFC is involved in the perception of nonverbal cues unique to humans. The OFC is attributed to human vs. non-human non-verbal expressions such as gestures. Participants were presented with video clips of gestures of emotional expressions or silent speeches impersonated by a human or a robot. They judged the emotional content or the amount of motion. The activity in the OFC was reduced when participants read angry emotions from the gestures of robots relative to humans [97]. These findings suggest that the robot did not elicit a desire in its interactant for social communication, which is sufficient to be reflected in OFC activity.

Secondly, the OFC is involved in recognizing expressive information in body language. Pictures displaying expressive body language (including facial, body expressions, and mimics) following verbal descriptions that were incongruent with the pictures showed increased N400, which was further localized in the ventromedial OFC [99]. The OFC can be related to the evaluation of expressive information, highlighting its role in processing bodily cues. The temporal OFC network is also associated with constructing meanings from body expression. A repetition suppression study showed reduced neural activity in bilateral MTG, STG, and OFC when expressive body movements were repeated in successive performances. Therefore, the temporal OFC networks mediate the lower-level representations of movement dynamics and socio-affective perceptual information to generate, evaluate, and update predictive inferences about expressive information.

Moreover, the OFC has been associated with detecting vocal cues associated with speaker identity. The OFC is associated with the top-down evaluation of emotionally relevant vocal information. In a task in which the participants were asked to identify the gender of the voice, the males showed stronger activity in the left mOFC than females in responding to the women’s voice relative to the men’s voice [98]. This finding suggests that the OFC may assign higher relevance to speech and vocal stimuli that are relevant to the listener. Neurophysiological measures using MEG showed that the early response at around 130 ms and late response at around 200 ms were linked with the differentiation of infant from adult crying, both localized in the OFC [100]. This supports more detailed processing of cue salience and meaning from infant vocal cues. The OFC may mediate the rapid allocation of attention to the infant’s vocal cues, and such a process can be modality-specific [142]. The OFC is associated with the paternal response towards children depending on the child’s gender. Compared with fathers of sons, fathers of daughters showed more robust neural responses towards their daughters’ happy expressions in the medial and lOFC. They used more analytical language and language related to sadness for their daughters. In contrast, fathers of sons showed increased response to their sons’ neutral expressions in the mOFC, and used more achievement language with their sons [101]. These findings suggest an important role of OFC in regulating emotional information in generating languages of different motivations.

#### 6.2.5. Interplay of Processing Language and Emotional Feelings

There was a negative association between the strength of low-frequency fluctuation in the OFC in the resting fMRI and trait hope and trait optimism in linguistic terms. The involvement of OFC in linguistic representation is against the proposal that language representation is isolated from perceptions, actions, or emotions and is a proponent of the embodied view that language is embodied and grounded in perception and action [143]. Indeed, the neural activity in the OFC is increased when participants read words that refer to their own emotions [144], highlighting the link between feelings of linguistic words and emotion perception. Visual exposure or reading emotionally laden words can cause (neuro)physiological changes in visual, sensorimotor, and even motivational processing of approach avoidance behaviors [145]. Similar findings are found for linguistic terms that express other feelings, such as hedonics [146].

The OFC regulates one’s emotional feelings across language tasks. The OFC is associated with the dispositional factors underlying anticipatory feelings (e.g., anticipation of reward), which can be verbally articulated in linguistic feeling terms [147].

### 6.3. Strategies for Using Socially Appropriate Languages

Successful communication in daily conversational exchanges recruits linguistic processes such as lexico-semantic processes and involves additional resources that ensure the socially appropriate use of language. Understanding or expressing a language in social contexts involves perspective taking or switching of one’s viewpoint on the theory of mind, perceiving negative consequences of using inappropriate language, and accessing the knowledge of the communicators. As shown in Table 3, enhanced activity in the OFC can be found when additional cognitive strategies or decisions are demanded to ensure the socially appropriate use of language.

The OFC was involved in theory of mind (TOM) processing, which is essential for social inference. Patients with OFC lesions showed a disrespectful use of language towards individuals with a higher social status. They exhibited uninhibited, frequent use of inappropriate linguistic behaviors, as well as endorsed forms of immoral actions that are rejected by healthy individuals [148,149,150].

The OFC may be associated with the perception of negative consequences in understanding pragmatic language. In the resting-state EEG recording, when asked why a friend refused their request, participants showed more significant activity localized in the OFC than when they merely thought of their friend [151]. The OFC is associated with the pragmatic inferences that involve decoding an interactant’s mental states during conversation. The comic strips that evoke the perceiver’s attribution of intention to the character activated the OFC compared to those that depict sequences of physical causality, as demonstrated in an fMRI study [152].

The OFC was associated with one’s ability to guide decisions towards a communicative partner. In a nonverbal communicative task where the participant aimed to inform the confederate of the location of a target object, subjects with lesions in the OFC spent disproportionally longer time on the locations of these objects, regardless of the presumed characteristics of the addressee (i.e., child vs. adult), which is different in communicative ability [153]. These findings suggest that the OFC plays a role in guiding people to fine-tune their communicative decisions to spontaneously adjust their speech and other nonverbal behaviors (gestures and body motions) when addressing a child, with their implied knowledge about the addressee.

The left OFC is involved in the mentalizing process during third-person sentence comprehension. Reading sentences with mental state words (e.g., persuade) gave rise to greater activation in the left OFC as compared with sentences with action verbs (e.g., punch), highlighting the role of the OFC in socio-communicative processes that are essential for decoding linguistic expressions [102]. Moreover, reading sentences of a story that demanded second-order false belief (e.g., A thinks B thinks) compared with reading unassociated sentences showed reduced activity in bilateral OFC. Moreover, such a reduction was more significant in females than in males [103]. These findings highlight the possible role of the OFC in indexing one’s pragmatic reasoning skills.

The involvement of the OFC in daily communicative performance is established in interactive tasks. Some studies conducted a referential communication task in which the participants and the interlocutors were presented with a visual array, and the participants were asked to identify a target object among competitors with their language using adjectives that correctly describe the target. The overuse or insufficient use of adjectives is not encouraged. The demand in shifting their perspective to the interlocutor is manipulated such that the demand is increased because some feature dimensions that distinguish between targets and competitors are not available to the interlocutor and are private to the participants [7]. In this task, a target that cannot be distinguished in color from the competitor from the interlocutor’s side would increase the perspective shifting demand for the participant when they had to refer to an alternative disambiguated feature, such as shape and size, which was expected for both sides. Structural MRI revealed decreased GMV in the OFC for behavioral variant frontotemporal dementia, which was correlated with poorer performance in the referential communication task (reflected by the inability to use more specified responses in the more demanding condition) and a visual–verbal test on mental set shifting. In accordance with the role of OFC in supporting the ability to adapt to new environmental contingencies and to reverse previously established stimulus reinforcement associations [154,155], the OFC here can be associated with the ability to adjust one’s strategy to use specified language in the referential communicative task [7]. One novel finding in this study was that the characteristics of the white matter tract, the uncinate fasciculus (UF), which connected the OFC to the anterior temporal lobe and was previously associated with both semantic [156,157] and social language processing [158,159], was correlated with a poorer performance in the referential communicative task [7]. This fasciculus of the OFC likely serves in daily communication by facilitating cross-talk between the social and language networks.

The OFC is sensitive to language that conveys a viewpoint that is congruent or incongruent with the perceiver’s own and may be involved in viewpoint switching. This was demonstrated in a study where participants were split into groups who underwent a speech construction task. They were asked to write a speech incongruent or congruent with their own viewpoint on a specific issue in the other (e.g., gambling). These tasks were followed by fMRI scanning, in which they were asked to judge another’s viewpoints on a contentious issue [104]. The OFC was more activated when they were asked to construct a speech from another’s (incongruent) viewpoint as compared with when they constructed one from their own. The right OFC activity was negatively associated with the individuals’ trait of stubbornness. The involvement in the speech construction task altered one’s prosocial-cognitive control and, in turn, the right OFC activity that the stubborn personality can mediate.

To sum up, under the primary role of the OFC in supporting the individual’s ability to adapt to new environmental contingencies and to reverse previously established stimulus reinforcement associations [160,161], the OFC is associated with the speaker or the listener’s strategy of targeting pragmatically felicitous and socially inappropriate use or interpretation of language, building connections between different modalities of linguistic and nonlinguistic forms and associating higher-level emotional meanings (including semantic representations, feelings and inferences) with lower-level linguistic inputs. These domain-general functions of the OFC, which guide a goal-directed language/speech comprehension or production, ensure the successful adaptation of the language user to the changing communicative settings during social communication.

## 7. Conclusions and Under-Explored Directions

### 7.1. Perisylvian Network and OFC: Division of Labor in Language Processing?

The above-reviewed studies either directly tested the neural activation of language or speech processing or tested language or speech-relevant performances in tasks and correlated them with the structural/functional characteristics of the brain. This review demonstrates that not only the linguistic tasks that involve the processing of socially, pragmatically, and emotionally relevant information engage the OFC and its neurobiological mechanisms, but also specific receptive and expressive language performances rely on specific neurophysiological properties of this region (e.g., the gray matter volume and the functional activation of the OFC and the UF that connects OFC), which in many cases, demand executive functions.

The perisylvian regions and OFC have been reported in many language and speech tasks, though the former network is assigned greater relevance to the language task per se. The evaluation of the activation coordinates showed that the OFC was coactivated with frontotemporal perisylvian regions in the main contrasts of conditions of interest regardless of the types of tasks or functions it played. The co-activation of OFC and perisylvian networks suggests that the OFC could serve an accessory or compensatory role in language/speech processing for the core language network.

However, beyond the review’s findings, one should further elucidate whether the division of labor could exist for these two separate neural correlates to support a certain linguistic function, even in the same task. For example, it has been suggested that multiple neural mechanisms may functionally support the semantic processes during language comprehension. While the frontotemporal network is essential in evaluating the linguistic expression against the context of one’s general world knowledge, the OFC can participate in the semantic composition processes, coercing a specific linguistic unit to others [106] or resolving referential ambiguity [63]. The unification and composition of linguistic units may seem to engage different labors.

Therefore, it is likely that other receptive or expressive language processes may also show such functional divisions of labor, such as visual word comprehension and speech production, in which both the left IFG and OFC have been reported. A neuroimaging study with a lexico-decision task showed that the perisylvian regions including the left IFG can dissociate between words preceded by both morphologically and semantically related lexical items and those preceded by morphologically related items only in a language with a linear morphology system (e.g., English) but not in a language with a rich and systematic morphological system (e.g., Hebrew). In contrast, the medial frontal gyrus seemed to be consistently activated on words preceded by morphologically irrelevant items rather than those preceded by related words, regardless of the language typological system [162]. These findings suggest that the perisylvian regions could serve linguistic processing functions in a language-dependent fashion, but the function of the OFC could be more language universal. Another interesting aspect regarding the functional dichotomy of perisylvian regions and the OFC is their differential capacity to predict differential problems in psychiatric or neurological patients. For example, in schizophrenic patients, the GMV in the frontal-temporal areas (e.g., STS/STG) predicted the severity of specific language disorders (e.g., semantic deficit), and the GMV in the OFC predicted disinhibition of self-relevant information [163]. Moreover, sex differences can also reveal which specific region is involved in the altered functional connectivity. During the first episode of schizophrenia, the aberrant functional connectivity with Broca’s area (BA 44 and 45) appeared in male patients, and the change in the connectivity pattern involving the orbital frontal gyrus was shown in female patients [164]. The functional segregations of the perisylvian regions and OFC can be disentangled by examining performances in tasks demanding more domain-general function or language/speech-specific functions on the same individuals to see if a pattern of task x region double dissociation can be clearly demonstrated.

### 7.2. OFC: Domain-Specific Language/Speech Processing vs. Domain-General Executive Process in Language/Speech Tasks That Involve Socio-Emotional Decisions?

There is an ongoing debate on the linguistic processes relevant to the perisylvian network whether the network is specific to human language functions. In a similar vein, it is unclear whether the OFC engaged in the processes mentioned above works as (1) a mechanism that is unique to language and speech processing and (2) a domain-general mechanism.

Before our review was conducted, some studies related the white matter connections of the OFC, the UF, which connects the OFC with the temporal limbic regions, with the specific aspect of linguistic processing, in particular, to encode, store, and retrieve semantic knowledge [165], rather than the general linguistic function. The UF has been considered part of the ventral language pathway and facilitates semantic naming by relaying sensory information about objects, presumably represented in the ventral temporal cortex, to language-supporting regions [166].

Our review of the involvement of OFC in different neuroimaging tasks appears to provide the support that OFC serves as a domain-general executive process in language/speech tasks that involve socio-emotional decisions. The OFC, if we take the semantic process as an example, executes a higher-level evaluative or decision-making process that allows for the integration of meanings in social contexts or performs executive functions that allow multiple meanings in ambiguity to be monitored, coordinated, and interactive with each other, like what medial PFC is typically expected to do [167]. Some studies speculate that UF participates in socio-language processes and is suggested to assign motivational values to pragmatically, socially and emotionally relevant information that is relevant to the language task. Von Der Heide et al. proposed that the UF functions in social language processing by allowing temporal lobe-based stimulus associations (e.g., name, face, voice, feelings about a person) to modify the communicator’s behavior via interactions with the OFC [166]. The interaction functions to assign motivational values to stored representations. The bi-directionality of UF information flow ensures that the stored representation reflects its most updated motivational values. This proposal allows for further investigation of the role of UF in recognizing nonverbal cues for interpersonal and communicative purposes. For example, patients with mere lesions in the OFC have shown impaired emotion recognition from tone of voice [168]. The OFC activity was enhanced when vocal cues that marked the speaker’s mental states were relevant to the listener’s evaluative task (judging the speaker’s confidence, [95,169]). In future studies, by relating the anatomical features of UF and the recognition accuracy in nonverbal cues, one can understand how the OFC functions to assign different motivational values to perceived nonverbal cues in language comprehension.

A possible model that considers both the perisylvian network and the OFC proposes several domain-general pathways that underlie decision making [3]. This model recruits the cortico-striatal pathways to general evaluative and selection processes across domains, including language production processes in linguistic decision-making. In particular, frontal language regions (BA44 and 45, which form Broca’s area, and BA47) connect to the caudate nucleus, premotor regions connect to the putamen, and the OFC connects to the ventral striatum. Different frontostriatal pathways are recruited to select an appropriate linguistic outcome from weighted options through a parallel mechanism to achieve efficient speech production. The caudate nucleus selects linguistic alternatives based on the “harmony” values (which possibly correspond to different linguistic constraints) assigned by frontal language areas, the putamen selects among motor plans based on weighted values assigned by premotor areas, and the ventral striatum selects among behavioral goals based on the reward/salience values assigned by the OFC (which corresponds to the evaluative goals in language communication; [3]). Supporting this model, evidence has shown that the strength of activation in the OFC and associated medial prefrontal regions in a nonverbal decision making task strongly predicts the longitudinal recovery of speech production after left-hemisphere stroke [51]. These processes seem to occur within a single domain when linguistic representation is simple and unimodal; however, it is unique to see how different pathways contribute to a complex and interactive setting that demands the selection of linguistic alternatives across different domains, e.g., multimodal speech production.

### 7.3. OFC and the Adaptive Processes in Language and Speech Communication

Our findings highlight that the OFC plays a relevant role in the adaptive neurobiological function of language. In particular, the neurobiological mechanisms beyond linguistic and speech processes complement and interplay with the language-unique processes to achieve successful comprehension and production in the changing communicative contexts.

The OFC has been predominantly associated with a function of stimulus–reinforcement association learning, a crucial function related to human adaptive behavior [46,160]. The OFC receives highly processed sensory information inputs, including those that encode bodily states and those from areas that process high-level emotional and social information. Crucially, neurons in OFC learn and reverse the stimulus to which they respond when the association of the stimulus with a primary reinforcing stimulus is reversed. The OFC generates outputs to the medial prefrontal cortex and medial striatum, allowing this region to encode associations between sensory stimuli in the external world and internal states and send signals to be further integrated into ongoing higher-order cognitive operations in other prefrontal regions.

Human language is evolved to encode unstable and changing events [1,25]. Utterances and vowels in spoken language are unfolded temporally, with auditory cues varying to express a rich set of meanings. Messages are not always specific or unambiguous, or they only encode a single meaning and are typically embedded in broader communicative contexts. Verbal and nonverbal signals sometimes conflict to convey nonliteral and indirect meanings. Turn-taking in conversations involves accurately predicting when the partner finishes their turn and when the speaker initiates their turn, with the expected and actual timings not aligned. Moreover, switching between languages of different morphological systems (e.g., from L1-Arabics to L2-Hebrew) or between languages of different social contexts or conversational scenarios of sociolinguistic varieties (e.g., diglossia; from a more informal spoken form to a more formal literary form) during language production often demands additional executive resources at both cognitive and speech-motor control levels [170]. Support for the diglossic language dominance emerged from a recent EEG study conducted by Khateb and Ibrahim (2022) [171]. Their research explored whether literary Arabic serves as a second language for native Arabic speakers and whether diglossia constitutes a unique form of bilingualism. The study revealed that native Arabic speakers proficient in both spoken and literary Arabic operate as if they possess two primary languages: one in the auditory form (spoken Arabic) and another in the visual written form (literary Arabic). The researchers discovered that, despite their competitive nature, the two forms exhibit similar behaviors during language production tasks. These findings bolster the argument that brain-based language dominance in a diglossic context is dependent on the modality of communication.

How do speakers and listeners monitor the changing status of learned language and communicative behaviors? How do comprehenders mentally simulate the consequences of unexpected linguistic or speech input not learned before? How do speakers effectively switch between languages according to the changing of social norms or communicative-pragmatic constraints? These unstable and changing events that occur in human language communication mean that language communicators must ensure the successful learning and reversal of linguistic stimulus–reinforcement associations, since previous reinforcement contingencies change. Such an evolving and changing nature of human language and speech has demanded future research that is dedicated to uncovering the role of the OFC and the neural circuit in goal-directed language comprehension and production behaviors.

Although a majority of studies on the neurobiology of language demonstrate the role of the perisylvian network in language and speech function, this review attempts to complement these pieces of evidence by focusing on the role of the OFC. This region has been primarily associated with social cognition. We showed that not only the speech and language processes that demand the use of social, pragmatic, and emotional information engage the OFC and its neurobiological mechanisms but also receptive and expressive linguistic processes that demand executive control rely on specific neurophysiological properties that involve this region. These findings highlight that (1) the neurobiological processes beyond those traditionally involved in language processing have a complementary role to language-unique processes to achieve successful language comprehension and production; (2) the OFC plays an non-negligible part for a speaker or a listener to adapt to a changing communicative environment.

## Figures and Tables

**Table 1:** Eligible neuroimaging studies on receptive language performance which showed activations in OFC. Peak activations are shown in MNI coordinates. Studies are listed following a chronological order. Tables and/or figures of source reference that showed the OFC activation are specified.

Reference	Peak Coordinate	Brain Region	Technique	Participant Language/Material Language	Tasks	Findings	Function
Nieuwland et al., 2007 (see Table 2 in [63])	(6, 66, −2)	Medial frontal	fMRI	Dutch/Dutch	Silent sentence comprehension of referential constituents	Referential ambiguity > Referential coherence	Semantic comprehension
	(−2, 62, 24)	Medial frontal	fMRI	Dutch/Dutch	Silent sentence comprehension of referential constituents	Referential ambiguity > Referential failure	Semantic comprehension
Diaconescu et al., 2011 (see Figure 7 in [64])	(−23, 41, −10) ^a^	OFC	MEG	English/English	Feature classification; cross-modal semantic congruency	Cross-modal congruency task > ^b^ Feature classification task towards the cross-modal display	Semantic comprehension
	(21, 41, −10) ^a^	OFC	MEG	English/English	Feature classification; cross-modal semantic congruency	Cross-modal congruency task > Feature classification task towards the cross-modal display	Semantic comprehension
McMillan et al., 2012 (see Table 2 in [65])	(−51, 33, −10)	orbitofrontal	fMRI	English/English	Probe verification of the referents of pronoun	Indirectly determined condition > Directly determined condition	Semantic comprehension
Kujala et al., 2007 (see Figure 4 in [66])	(−9, 28, 21) ^a^	OFC	MEG	English/English	Text reading comprehension	Increased activation as a function of the increasing speed of RSVP	Reading
Wang et al., 2019 (see SM Table 7 in [67])	(10, 56, −4)	bilateral orbital frontal cortex	fMRI	English/English	Passage reading comprehension followed by an independent N-back task	Increased activation as a function of the reader’s decreased executive ability	Reading
	(−10, 54, 8)	bilateral orbital frontal cortex	fMRI	English/English	Passage reading comprehension followed by an independent N-back task	Increased activation as a function of the reader’s decreased executive ability	Reading
Sabri et al., 2008 (see Table 1 in [68])	(−14, 32, 1) ^a^	left OFC	fMRI	English/English	One-back matching judgement on two consecutive trials while attentional relevance was manipulated	Attended sound > Ignored sound	Speech perception
Hsu et al., 2014 (see Figure 5 in [69])	(27, 21, −4) ^a^	right ventral-orbital frontal cortex	MEG	Mandarin/Mandarin	Passive watching of a silent movie while listening to the auditory stimuli sequence (auditory oddball paradigm)	Large-deviant tone (tone 1) > Standard tone (tone 3)	Speech perception

Notes: ^a^: indicates that the original coordinate in reference paper is in Talairach coordinate system and was transformed to the MNI coordinate in the table; ^b^: “>” represents the increased activation for the left compared to the right condition; fMRI = functional magnetic resonance imaging; MEG = magnetoencephalography; MNI = Montreal Neurological Institute; OFC = orbitofrontal cortex; SM = Supplementary Materials. Peak coordinates are visualized in Figure 1.

**Table 2:** Eligible neuroimaging studies on expressive language performance which showed activations in OFC. Peak activations are shown in MNI coordinates. Studies are listed following a chronological order. Tables and/or figures of source reference that showed the OFC activation are specified.

Reference	Peak Coordinate	Brain Region	Technique	Participant Language/Material Language	Tasks	Findings	Function
Nathaniel-James et al., 2002 (see Table 3 in [70])	(28, 28, −28)	Right oribital	fMRI	English/English	Sentence completion task with different instructions (initiation: generating congruent words or suppression: generating	Low contextual constraint > high contextual within the initiation task	Control in language production
Allen et al., 2008 (see Table 2 in [71])	(−24, 32, −18)	orbital gyrus	fMRI	English/English	Sentence completion task with different instructions (initiation: generating congruent words or suppression: generating incongruent words)	Suppression > ^b^ initiation	Control in language production
Hsieh et al., 2009 (see Table 2 in [72])	(23, 10, 15) ^a^	OFC	fMRI	English/English	Driving task only or driving task while a simulated hands-free cellular conversations task	Long conversation > no conversation	Control in language production
	(−30, 26, 5) ^a^	OFC	fMRI	English/English	Driving task only or driving task while a simulated hands-free cellular conversations task	Long conversation > no conversation	Control in language production
Delshad et al., 2017 (see Table 2 in [73])	(44, 24, −6)	right frontal orbital cortex	fMRI	Persian/Persian	Visual confrontation naming (covert picture naming)	visual confrontation naming task > rest task	Visual confrontation naming
Graves et al., 2019 (see Table 1 in [74])	(−9, 30, −9) ^a^	left medial OFC	fMRI	American English/American English	Reading aloud letter strings	Non-words > words (gender x age interaction)	Reading aloud
Montani et al., 2019 (see Table 2 in [75])	(−10, 36, −12)	medial orbitofrontal	EEG-source localization	French/French	Reading aloud words and pseudowords	High > low-frequency words	Reading aloud

Notes: ^a^: indicates that the original coordinate in reference paper is in Talairach coordinate system and was transformed to the MNI coordinate in the table; ^b^: “>” represents the increased activation for the left than the right condition; EEG = electroencephalograph; fMRI = functional magnetic resonance imaging; MNI = Montreal Neurological Institute. Peak coordinates are visualized in Figure 2.

**Table 3:** Eligible neuroimaging studies on processing pragmatically, socially and emotionally relevant information in language communication which showed activations in OFC. Peak activations are shown in MNI coordinates. Studies are listed following a chronological order. Tables and/or figures of source reference which showed the OFC activation are specified.

Reference	Peak Coordinate	Brain Region	Technique	Participant Language/Material Language	Tasks	Findings	Function
Maguire et al., 1999 (see Table 5 in [76])	(1, 39, −12) ^a^	ventromedial orbitofrontal region (BA11)	fMRI	English/English	Multimodal story listening followed by comprehensibility rating	Increased activation as a function of increased comprehensibility	Processing multimodal information
Goldberg et al., 2006 (see Table 1 in [77])	(−19, 33, −10) ^a^	left OFC	fMRI	American English/English	Semantic decision of sensory modality of words	Words in gustatory modality > pseudowords	Processing multimodal information
McNamara et al., 2008 (see Table 1 in [78])	(36, 39, −12)	orbital frontal gyri	fMRI	English/English	Associative learning of sound and gesture pairs; testing with the sound-gesture matching	Negative correlation of activation in sound–gesture matching and that in associative learning	Processing multimodal information
Barros-Loscertales et al., 2011 (see Table 2 in [79])	(−39, 33, −15)	frontal operculum/lateral OFC	fMRI	Spanish/Spanish	Passive comprehension of words	Taste-related words > words with few gustatory semantic links	Processing multimodal information
Olofsson et al., 2014 (see Figure 5A in [80])	(38, 32, −14)	right central OFC	fMRI	English/English	Sensory cue–lexical target matching task	Mismatching > matching odor-related words	Processing multimodal information
Pomp et al., 2018 (see Table 4 in [43])	(−27, 39, −15)	left medial inferior orbitofrontal gyrus	fMRI	German/German	Silent sentence reading followed by comprehension questions	Olfactory metaphors > literal paraphrases	Processing multimodal information
	(−46.5, 34.5, −9)	left inferior orbitofrontal gyrus	fMRI	German/German	Silent sentence reading followed by comprehension questions	Literal olfactory sentences (olfactory words serve literal meanings) > literal paraphrases	Processing multimodal information
Ferstl et al., 2005 (see Table 3 in [81])	(1, 18, −5) ^a^	ventromedial prefrontal cortex	fMRI	German/German	Judging the consistency of the stories of different types	Stories with emotional information > non-emotional condition	Understanding emotional meanings in and with language: affective consequences in non-literal and narrative language
Harada et al., 2009 (see Table 3 in [82])	(−6, 46, 40)	posterior rostral medial frontal cortex	fMRI	Japanese/Japanese	Judging a story on whether the protagonist told a lie, or on the morality of the behavior, or the protagonist’s gender	Lie judgment > gender judgment	Understanding emotional meanings in and with language: affective consequences in non-literal and narrative language
	(−8, 44, 46)	posterior rostral medial frontal cortex	fMRI	Japanese/Japanese	Judging a story on whether the protagonist told a lie, or on the morality of the behavior, or the protagonist’s gender	Moral judgment > gender judgment	Understanding emotional meanings in and with language: affective consequences in non-literal and narrative language
Wallentin et al., 2011 (see Table 3 in [83])	(−12, 60, −4)	medial orbitofrontal	fMRI	Danish/Danish	Listening to the story and detecting a target voice	Positively valenced > negatively alenced story	Understanding emotional meanings in and with language: affective consequences in non-literal and narrative language
Nummenmaa et al., 2014 (see SM Table 2 in [84])	(−10, 52, −6)	OFC	fMRI	Finnish/Finnish	Listening to narratives and imagined the events followed by a post-fMRI independent valence rating	Increased activation as a function of valence rating	Understanding emotional meanings in and with language: affective consequences in non-literal and narrative language
Lehne et al., 2015 (see Table 1 in [85])	(48, 20, 22)	medial frontal cortex	fMRI	German/German	Reading literary texts and rating the degree of suspense they experienced	Increased activation as a function of suspense rating	Understanding emotional meanings in and with language: affective consequences in non-literal and narrative language
Chan & Lavallee, 2015 (see Table 6 in [86])	(−30, 41, −11)	OFC	fMRI	Mandarin/Mandarin	Rating the funniness of jokes of different types	Bridging inference jokes > non-funny stories	Understanding emotional meanings in and with language: affective consequences in non-literal and narrative language
Ethofer et al., 2006 (see Table 1 in [87])	(−48, 30, −6)	left orbitofrontal gyrus	fMRI	German/German	Judge the valence of semantics or valence of the affective prosody of the emotional word	Semantic valence task > Affective prosody task	Understanding emotional meanings in and with language: processing emotional connotation in words
Alia-Klein et al., 2007 (see Figure 3 in [88])	(27, 29, 5) ^a^	right lateral and posterior aspect of OFC	fMRI	English/English	Lexical detection	Negative association of negative valence of “No” vs. “UP” condition and neural activity to “No” vs. “UP” at participant level	Understanding emotional meanings in and with language: processing emotional connotation in words
	(30, 27, −1) ^a^	right OFC	fMRI	English/English	Lexical detection	Positive association of emotional control of anger and neural activity to “No” vs. baseline at participant level	Understanding emotional meanings in and with language: processing emotional connotation in words
Keuper et al., 2013 (see Table 1 in [89])	(30, 60, −11)	right orbital middle frontal	MEG	German/German	Covert reading of words	Positive > negative words	Understanding emotional meanings in and with language: processing emotional connotation in words
Hinojosa et al., 2015 (see Figure 5 in [90])	(45, 55, −5)	OFC	EEG-source localization	Spanish/Spanish	Digit categorization with distractor words	Positive > neutral distractor words	Understanding emotional meanings in and with language: processing emotional connotation in words
Callan et al., 2006 (see Table 2 in [91])	(−2, 42, −6) ^a^	OFC	fMRI	Japanese/Japanese	Passive listening to singing; passive listening to speech; covert production of songs; covert production of speeches	Listening to singing > listening to speech	Understanding emotional meanings in and with language: perceiving emotional tone of voice
Ethofer et al., 2009 (see Table 2 in [92])	(54, 30, −9)	right OFC	fMRI	German/German	Judging the affective prosody (angry, neutral) or word class (adjective, noun) according to requirements	Angry > neutral prosody	Understanding emotional meanings in and with language: perceiving emotional tone of voice
	(−42, 30, −15)	left OFC	fMRI	German/German	Judging the affective prosody (angry, neutral) or word class (adjective, noun) according to requirements	Angry > neutral prosody	Understanding emotional meanings in and with language: perceiving emotional tone of voice
Mitchell, 2013 (see Table 1 in [93])	(18, 35, −6) ^a^	medial OFC (vmPFC)	fMRI	English/English	Judging the emotion of prosody from words of matching or mismatching association of lexico-semantic valence and emotional prosody	Incongruent > congruent cues	Understanding emotional meanings in and with language: perceiving emotional tone of voice
	(−50, 25, 9) ^a^	lateral OFC (vlPFC)	fMRI	English/English	Judging the emotion of prosody from words of matching or mismatching association of lexico-semantic valence and emotional prosody	Incongruent > congruent cues	Understanding emotional meanings in and with language: perceiving emotional tone of voice
Peron et al., 2016 (see Table 1 in [94])	(21, 46, −10)	right orbital gyrus	fMRI	French/French	Judging the emotional prosody (angry, neutral) or the gender of voice (male, female)	Increased activity in the functional connectivity of subthalamas nucleus for emotional vs. neutral voices during gender task	Understanding emotional meanings in and with language: perceiving emotional tone of voice
Jiang et al., 2017 (see Table 3 in [95])	(−16, 36, −6)	left medial orbital frontal gyrus	fMRI	English/English	Speaker believability judgment towards vocal expression	Prosodically marked > unmarked expression	Understanding emotional meanings in and with language: perceiving emotional tone of voice
Zhang et al., 2018 (see Table 1 and Figure S1D in [96])	(−32, 62, −8)	orbitofrontal area	fNIRs	Mandarin/Mandarin	Passive listening of emotional pseudosentences	Pseudosentences with angry prosody > those of neutral prosody	Understanding emotional meanings in and with language: perceiving emotional tone of voice
	(35, 63, −8)	frontopolar area	fNIRs	Mandarin/Mandarin	Passive listening of emotional pseudosentences	Pseudosentences with angry prosody > those of neutral prosody	Understanding emotional meanings in and with language: perceiving emotional tone of voice
Chaminade et al., 2010 (see Table 2 in [97])	(28, 40, −4)	right middle orbital gyrus	fMRI	English/English	Rating the emotional content or amount of motion from human or robot expressions	Human angry expression > robot angry expression	Understanding emotional meanings in and with language: perceiving salient nonverbal cues
Junger et al., 2013 (see Table 2 in [98])	(−12, 35, −14)	Medial OFC	fMRI	German/German	Judging the gender of voice	Female > male voices in men	Understanding emotional meanings in and with language: perceiving salient nonverbal cues
Proverbio et al., 2014 (see Table 3 in [99])	(1, 35, −8) ^a^	medial frontal gyrus	EEG-source localization	English/English	Verifying congruency between verbal descriptions and pictures	Congruent > incongruent	Understanding emotional meanings in and with language: perceiving salient nonverbal cues
Young et al., 2016 (see Table 2 in [100])	(−34, 20, −16)	OFC	MEG		Listening to vocalizations and detecting pure tones	Infant cry > adult cry	Understanding emotional meanings in and with language: perceiving salient nonverbal cues
Mascaro et al., 2017 (see Table 3 in [101])	(−13, 20, −5) ^a^	left medial orbital gyrus	fMRI	English/English	Viewing and sharing emotions of faces in photographs	Own son’s neutral face > Own daughter’s neutral face in fathers	Understanding emotional meanings in and with language: perceiving salient nonverbal cues
Kana et al., 2012 (see Table 1 in [102])	(−12, 46, −12)	frontal medial orbital	fMRI	English/English	Sentence reading followed by probe verification	Sentence with mental state words > ^b^ sentence with action words	Strategies of using socially appropriate languages
Frank et al., 2015 (see Table 2 in [103])	(−12, 58, 28)	mPFC	fMRI	English/English	Reading and continuing the story with the appropriate continuation	False-belief story > unlinked sentences	Strategies of using socially appropriate languages
Miura et al., 2020 (see Table 2 in [104])	(38, 46, 4)	right middle orbital gyrus	fMRI	Japanese/Japanese	Judging another person’s viewpoint statement in the scanner preceded by a speech writing task outside the scanner	Writing speeches inconsistent with one’s perspective > writing consistent speeches during viewpoint judgment	Strategies of using socially appropriate languages

Notes: ^a^: indicates that the original coordinate in reference paper is in Talairach coordinate system and was transformed to the MNI coordinate in the table; ^b^: “>” represents the increased activation for the left than the right condition; EEG = electroencephalograph; fMRI = functional magnetic resonance imaging; fNIRS = functional near-infrared spectroscopy; mPFC = medial prefrontal cortex; MEG = magnetoencephalography; MNI = Montreal Neurological Institute; OFC = orbitofrontal cortex; vlPFC = ventrolateral prefrontal cortex; vmPFC = ventromedial prefrontal cortex; SM = Supplementary Materials. Peak coordinates are visualized in Figure 3 and Figure 4.
